# Regulation of MicroRNA Biogenesis: A miRiad of mechanisms

**DOI:** 10.1186/1478-811X-7-18

**Published:** 2009-08-10

**Authors:** Brandi N Davis, Akiko Hata

**Affiliations:** 1 Department of Biochemistry, Tufts University School of Medicine, Boston MA 02111, USA; 2 Molecular Cardiology Research Institute, Tufts Medical Center, Boston, MA 02111, USA

## Abstract

microRNAs are small, non-coding RNAs that influence diverse biological functions through the repression of target genes during normal development and pathological responses. Widespread use of microRNA arrays to profile microRNA expression has indicated that the levels of many microRNAs are altered during development and disease. These findings have prompted a great deal of investigation into the mechanism and function of microRNA-mediated repression. However, the mechanisms which govern the regulation of microRNA biogenesis and activity are just beginning to be uncovered. Following transcription, mature microRNA are generated through a series of coordinated processing events mediated by large protein complexes. It is increasingly clear that microRNA biogenesis does not proceed in a 'one-size-fits-all' manner. Rather, individual classes of microRNAs are differentially regulated through the association of regulatory factors with the core microRNA biogenesis machinery. Here, we review the regulation of microRNA biogenesis and activity, with particular focus on mechanisms of post-transcriptional control. Further understanding of the regulation of microRNA biogenesis and activity will undoubtedly provide important insights into normal development as well as pathological conditions such as cardiovascular disease and cancer.

## Introduction

microRNAs (miRNAs) have been reported to control diverse aspects of biology, including developmental timing, differentiation, proliferation, cell death, and metabolism [1-3]. Because miRNAs exert these functions primarily through the repression of target genes, the determination of miRNA targets has been an area of intense research. Computational and experimental approaches indicate that a single miRNA may target several dozen or even hundreds of mRNA [4]. Additionally, more than 60% of human protein coding genes are predicted to contain miRNA binding sites within their 3' untranslated regions (UTRs) [4,5]. The diversity and number of miRNAs suggests that a vast number of normal and pathological outcomes may be controlled, at least in part, through miRNA-mediated repression. While some miRNAs are described as regulating developmental switches mediated through the repression of a single target, it is increasingly clear that the majority of miRNAs exert their effects through the modest reduction of a large number of targets which altogether give alterations in cellular phenotype [4,6].

miRNAs select mRNA targets for down-regulation through the association with a large, multi-protein complex, the RNA Induced Silencing Complex (RISC). This selection requires the presence of sequences within the target mRNA which are imperfectly complementary to the miRNA sequence; miRNA binding sites commonly occur within the 3'-untranslated region (UTR) of the mRNA, but functional miRNA binding sites can also occur with the 5'UTR [7] or coding region [8]. Intriguingly, the presence of a miR-148 binding site within an alternatively-spliced exon of DNA (cytosine-5-)-methyltransferase 3 beta (Dmnt3b) can promote the differential expression of splice variants [9].

The absence of perfect complementarity between the miRNA sequence and target sites complicates the identification of miRNA target genes. However, Watson-Crick base pairing between the target and the 5'-end of miRNAs, particularly the 'miRNA seed' (comprised of nucleotides 2–7), is an important determinant of functional target sites [10]. Given that the entire 22 nucleotide (nt) sequence of many miRNAs is conserved throughout evolution, it is highly likely that the 3'-region of miRNAs also plays a critical role in the determination of miRNA targets [10,11]. Although the identification of miRNA targets has been enabled by computational approaches, it remains unclear what determines the degree of repression of a given miRNA-target mRNA pair.

The precise mechanism of miRNA-mediated gene regulation has been a subject of intense research [12]; miRNAs have been reported to repress the translation of target mRNA by blocking translation initiation and/or elongation [13-16]. Alternatively, targets of miRNA may be sequestered away from the translation machinery and transferred to processing-bodies (p-bodies), where mRNAs are known to be degraded [17]. The precise mechanism of miRNA-mediated translational repression varies depending on the specific system of study, and is reviewed in detail elsewhere [12]. Although early work with let-7 suggested that miRNAs act primarily at the level of translation without altering mRNA levels [13], recent genome-wide profiling experiments indicate that most highly repressed miRNA targets exhibit some degree of mRNA degradation [6]. miRNA interaction may promote the destabilization of target mRNAs through the recruitment of decapping and deadenylation complexes [18,19]. Alternative mechanisms of mRNA repression may be utilized depending on the cellular context, RNA secondary structure, or effector proteins associated with a specific miRNA:mRNA pair [20]. For example, transcription of a miRNA from different promoters altered the specific mechanism of repression; suggesting that the nuclear history of a miRNA may influence silencing [21]. The further clarification of the mechanisms of miRNA-mediated silencing and the requirements of functional miRNA target sites is an area of active research and will be important in understanding the physiological role of miRNA in diverse areas.

## Spatiotemporal regulation of miRNA expression

The founding members of the miRNA family, lin-4 and let-7, were originally identified in *C. elegans *and described as small temporal RNAs (stRNA) due to their specific pattern of expression during development [22,23]. Amazingly, the temporal regulation of let-7 miRNA was conserved through humans, suggesting that miRNAs could be expressed in defined spatiotemporal patterns [24]. This observation has been further extended as the expression profiles of more miRNAs have been determined. miRNA profiling in mouse and human adult organs indicated ~50% of miRNAs are expressed in a tissue-specific manner [25,26]. For example, cloning of small RNAs from different mouse tissues identified miR-1, miR-122a, and miR-124 as highly enriched in the heart, liver, and brain, respectively [27]. *In situ *hybridization of zebrafish embryos identified a large percentage of tissue-specifically expressed miRNAs which are also temporally regulated during development [28]. Furthermore, increased expression of tissue-specific miRNAs (ie: miR-124 in neurons, let-7 in differentiated cells) represses progenitor cell-specific mRNA to increase the fidelity of cell fate transition during differentiation [29,30].

Given the importance of miRNAs in development, it is not surprising that deregulation of miRNA expression is implicated in a variety of human diseases, including cancer and heart failure. Many miRNAs are located at sites of genomic instability, including duplications and fragile sites [31]. Global miRNA expression is frequently reduced in tumor samples relative to normal tissues, suggesting a role for miRNA in maintaining differentiated cell phenotypes. Indeed, let-7 expression is often dramatically reduced in lung cancer and exogenous expression of let-7 can dramatically inhibit tumor growth *in vivo *[32]. Conversely, a subset of miRNAs, including miR-21 and miR-155, have been identified as highly expressed in a variety of tumors and may serve to promote tumor growth through the inhibition of pro-apoptotic pathways. miRNA expression profiling serves as a better predictor of tumor origin and prognosis than conventional gene arrays, further emphasizing the importance of miRNAs in oncogenesis [33]. For example, a recent miRNA profiling study of pancreatic tissues indicates that the expression of as few as 25 miRNAs could distinguish among pancreatic adenocarcinoma, chronic pancreatitis, and normal pancreatic tissue [34,35]. Furthermore, the pattern of miRNA expression serves as a highly reliable tool to predict therapeutic outcomes in leukemia and colon cancer [36,37]. Understanding the mechanisms that control both normal and deregulated miRNA expression may lead to new avenues for treatment of a variety of disorders.

## Overview of miRNA biogenesis

**Figure 1:**
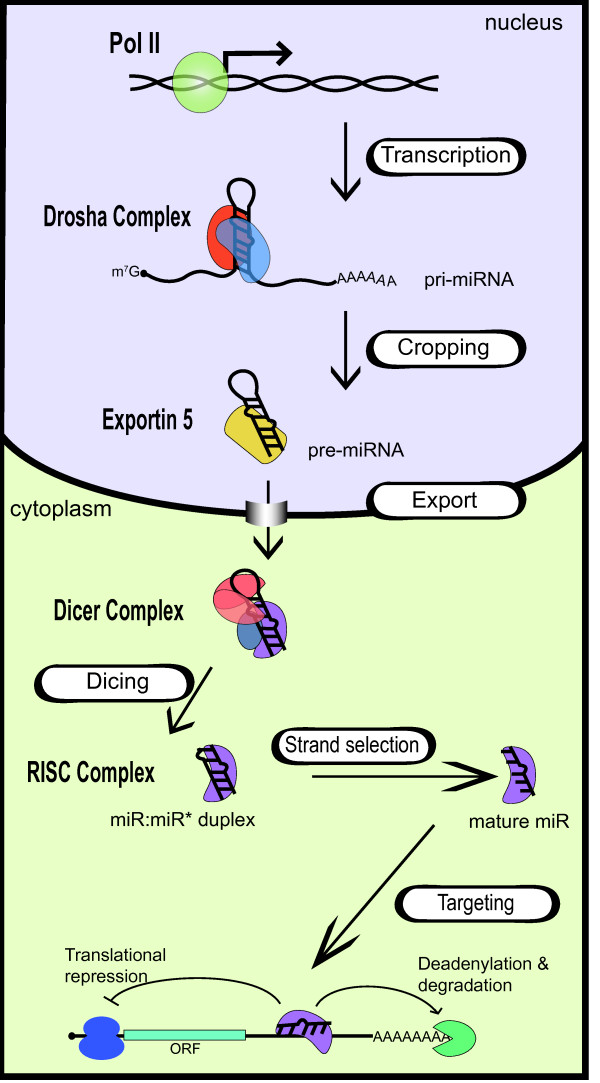
**Biogenesis of miRNAs and assembly into RISC complex**. RNA pol II generates capped and polyadenylated pri-miRNAs which are processed by Drosha in the nucleus to generate pre-miRNAs. After translocation into the cytoplasm by exportin 5, pre-miRNAs are processed by Dicer to form the mature miRNA/miRNA* duplex. Following processing, miRNAs are assembled into the RISC complex. Only one strand of the duplex is stably associated with the RISC complex. The mature miRNA directs repression of mRNA containing partially complementary miRNA binding sites within the 3'UTR.

The evolutionarily-conserved mechanism which gives rise to mature miRNA involves two ordered endonucleolytic cleavages by the RNase III enzymes Drosha and Dicer (Fig. 1) [38]. Following transcription by RNA polymerase II (RNA pol II), Drosha processes the primary miRNA transcript (pri-miRNA) into a ~60–100 nt hairpin structure termed the precursor-miRNA (pre-miRNA) [39-42]. Through the interaction with exportin-5 and Ran-GTP, the pre-miRNA is transported into the cytoplasm, where it undergoes a second round of processing catalyzed by Dicer (Fig. 1) [43,44]. This cleavage event gives rise to a double-stranded ~22 nt product comprised of the mature miRNA guide strand and the miRNA* passenger strand. The mature miRNA is then loaded into the RISC while the passenger strand is degraded (Fig. 1) [45]. Although major progress has been made in understanding the basic mechanism of miRNA biogenesis, less is known about the specific mechanisms that regulate miRNA expression. As discussed below, each step of the general biogenesis pathway has been found to be differentially regulated to allow exquisite control of miRNA expression. Recent studies have revealed that all miRNAs are not created equally and different mechanisms allow for the specific control of individual miRNAs.

## Genomic organization and transcription of miRNA genes

miRNAs are encoded in diverse regions of the genome including both protein coding and non-coding transcription units. Approximately 50% of miRNAs are derived from non-coding RNA transcripts, while an additional ~40% are located within the introns of protein coding genes [46,47]. The majority of miRNAs are transcribed by RNA polymerase (RNA pol) II and bear a 7-methyl guanylate cap at the 5' end and poly (A) tail at the 3' end, similar to mRNAs (Fig. 1) [39,48]. RNA pol III has also been demonstrated to generate the transcripts of a subset of miRNAs [49,50]. Co-regulation of mRNA and miRNA expression can be achieved by embedding the miRNA sequence within the intron of protein coding genes, or occasionally within the 3'UTR of a mRNA [47]. Furthermore, some miRNAs present in the 3' flanking regions of coding genes may be generated by read-through transcription [51].

**Figure 2:**
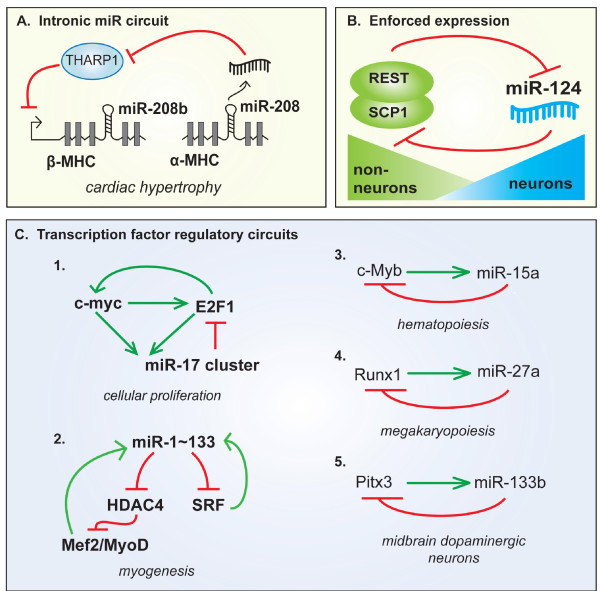
**miRNA regulatory circuits**. **A**. The cardiac specific miR-208 family is encoded within the introns of myosin heavy chain (MHC) genes. miR-208 targets THARP1, which then down regulates the expression of β-MHC gene. **B**. Expression of miR-124 is negatively regulated by the binding of the RE1 silencing transcription (REST) factor to the promoter in non-neuronal cells. **C**. Examples of feed-back regulation of microRNA transcription through the repression of transcription factors.

Drosha cleavage of intronic pri-miRNA precedes splicing, and the insertion of a miRNA hairpin into an intron does not alter host gene splicing or stability, indicating that a single transcript could give rise to both miRNA and mRNA [48,51,52]. Additionally, in some cases, the presence of a miRNA within an intron may enhance processing of the miRNA by extending the time of pri-miRNA association with chromatin [53]. An important role for intronic miRNAs is highlighted by the muscle specific miRNAs (or myo-miRs) miR-208, -208b and -499, which are encoded within the introns of myosin heavy chain (MHC) genes (Fig. 2A) [54]. MHC genes are specifically expressed in cardiac muscle and therefore drive the tissue-specific expression of myo-miRs. Furthermore, the expression of α-MHC and β-MHC is reciprocally regulated during cardiac remodeling following injury (Fig. 2A). *In vivo *evidence suggests that miR-208 encoded within the α-MHC gene utilizes diverse signaling mechanisms to control expression of the miR-208b host gene, β-MHC [55].

The presence of miRNA within an intron does not necessitate co-regulation of the miRNA with the host gene. Microarray analysis of miRNA expression of 175 miRNAs in 24 human organs found just 16 of 34 intronic miRNAs that were strongly co-expressed with the host gene [56]. The difference in host gene and mature miRNA expression could occur through differential miRNA processing or through alternative promoter usage. Indeed, nucleosome mapping studies and RNA pol II chromatin immunoprecipitation studies indicate that 25–33% of intronic miRNAs are transcribed from independent promoters [50,57].

Many miRNAs are encoded in the genome as clusters which can range from 2 to 19 miRNA hairpins encoded in tandem in close proximity. Detection of polycistronic transcripts containing multiple miRNAs by Northern blot suggests that clustered miRNAs can derive from a single transcript [56]. However, it is clear that clustered miRNAs are not necessarily co-regulated [25,58]. While some clustered miRNAs can arise from independent transcripts, as in the case of miR-433 and miR-127 [59], differential post-transcriptional processing of clustered miRNAs has also been observed, as in the case of miR-18a [60]. Additionally, in some instances, a single miRNA locus can give rise to two miRNAs with distinct seed sequences through bidirectional transcription [61,62].

## Regulation of miRNA expression: Transcription

RNA pol II mediated transcription provides a major control point for the biosynthesis of miRNAs. A recent large scale mapping of 175 human miRNA promoters through nucleosome positioning and chromatin immunoprecipitation-on-genomic DNA microarray chip (or ChIP-on-chip) analysis suggests that the promoter structure of miRNA genes, including the relative frequencies of CpG islands, TATA box, TFIIB recognition, initiator elements, and histone modifications, is indistinguishable between the promoters of miRNA and mRNA [50,57]. Furthermore, DNA binding factors that regulate miRNA transcription largely overlap with those that control protein coding genes.

### c-Myc: a critical regulator of miRNA transcription

**Figure 3:**
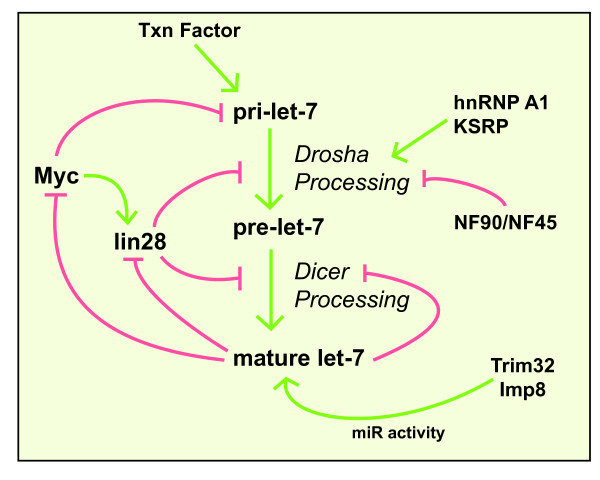
**Intricate network of regulation of let-7 expression and activity by different proteins**. Multiple mechanisms give rise to an intricate feed-back loop which controls the expression of let-7 and Lin-28. Lin28, which is highly expressed in undifferentiated, embryonic stem cells, selectively blocks the processing of let-7 miRNAs through multiple mechanisms including Drosha blockade, Dicer blockade, and 3'-uridylation of pre-let-7. The highly regulated expression of let-7 is critical for the control of cellular differentiation and proliferation.

The proto-oncogene c-Myc is a helix-loop-helix leucine zipper transcription factor which regulates ~10 to 15% of human genes to control cell growth and apoptosis [63]. Deregulation of c-Myc is a hallmark of many cancers and is associated with both activation and repression of transcription of protein coding genes [64]. It is now clear that c-Myc also modulates transcription of miRNA genes [65,66]. c-Myc binds to E-boxes in the promoter and activates transcription of the miR-17-92 cluster [65]. Consistent with the prominence of c-Myc activation in tumors, miR-17-92 is often highly expressed in tumors. The miR-17-92 cluster contains 6 miRNAs, including miR-17-5p and miR-20, which are known to repress the cell cycle regulator E2F1 [65]. Interestingly, c-Myc also promotes E2F1 transcription; suggesting that c-Myc is able to fine tune cell cycle progression via the regulation of both miRNA and mRNA expression (Fig. 2C) [67]. In addition to activating transcription, c-Myc also decreases the expression of several tumor suppressor miRNA genes, including the miR-15a, -29, -34 and let-7 families [66]. Exogenous expression of c-Myc-repressed miRNAs in lymphoma cells reduced cell growth, indicating that down regulation of a subset of miRNAs is an important mechanism of c-Myc-mediated tumorigenesis [66]. ChIP analysis reveals that this repression is mediated, at least in part, through direct binding of c-Myc to miRNA promoters [66]. Interestingly, c-Myc has also been shown to increase transcription of Lin-28B which in turn mediates the post-transcriptional repression of let-7 family members (Fig. 3 and discussion below) [68]. Consistent with the repression of let-7 by c-Myc, increased expression of c-Myc in Burkitt lymphoma cells is associated with decreased let-7 expression [69]. *c-myc *is also targeted by let-7, suggesting a double negative feed-back loop in which c-Myc represses let-7 transcription and let-7 in turn represses c-Myc (Fig. 3) [69]. Together these studies provide just one example of the dynamically regulated transcriptional control of miRNAs and are illustrative of the biological outcomes which are mediated by altered transcription of miRNA genes. c-Myc is not alone in this arena: the tumor suppressor p53 activates transcription of the miR-34 family, which in turn promotes cell cycle arrest and apoptosis [70,71].

### miRNA transcription networks

The tissue-specific expression of protein coding genes is controlled by complex transcription factor networks which orchestrate the differentiation and maintenance of cell fate in specialized tissues. Consistently, the transcription of tissue-specific miRNAs is often modulated by the same master regulatory factors which regulate mRNA. For example, skeletal and cardiac muscle differentiation is characterized by the transcriptional activation of muscle specific genes through the cooperation of serum response factor (SRF) and myocyte enhancer factor 2 (MEF2) with muscle-specific basic-helix-loop-helix transcription factors, including Myf-5, MyoD, and Myogenin [72]. SRF and MEF2 have recently been described to also promote the transcription of several myocyte-specific miRNAs, including miR-1, -133, -206, and -208 (Fig. 2C) [54]. Similar to myogenesis, the regulation of haematopoetic cell differentiation is controlled by combinations of lineage-specific transcription factors. miR-223 is highly expressed in the myeloid cell lineage and is activated by the myeloid transcription factors, C/EBP and PU.1 [73]. The coordinated expression of tissue specific miRNAs with mRNAs may serve to enforce cell fate switching during development. Negative regulation of miRNA transcription has also evolved to prevent misexpression of cell type-specific miRNAs. For example, expression of miR-124, which is abundant in brain, is negatively regulated by the binding of the RE1 silencing transcription (REST) factor to the promoter (Fig. 2B) [74]. REST and its cofactor Small C-terminal domain phosphatase 1 (SCP1) are highly expressed in neuronal precursors and non-neuronal genes. The REST complex silences transcription of neuronal genes through the recruitment of histone deacetylases (HDACs) and the methyl CpG binding protein MeCP2 [75]. Therefore, the REST complex prevents the misexpression of neuronal genes, including miR-124, outside of the nervous system. The interplay between the REST complex and miR-124 sets up a feedback loop which stabilizes and enforces the expression of neuronal vs. non-neuronal genes (Fig. 2B) [74,76]. Given that exogenous expression of miR-124 in HeLa cells down-regulates hundreds of non-neuronal targets, the tight control of miR-124 expression is critical for the diversification of neuronal from non-neuronal cells [77].

Bioinformatic analyses indicate that miRNAs may have a general propensity to regulate transcription factors [78]. Interestingly, an emerging theme in tissue-specific miRNA regulation is that miRNAs are often involved in complex regulatory networks with the transcription factors that drive their expression. Autoregulation of miRNA expression is found when the transcription factor which regulates miRNA expression is targeted by the miRNA itself. Examples of this type of regulation include Runx1-miR-27a in megakaryopoiesis [79], Pitx3-miR-133b in midbrain dopaminergic neurons [80] and c-Myb-miR-15a in hematopoiesis (Fig. 2C) [81]. This type of regulation allows tight control of miRNA and transcription factor levels. Feed-forward circuits have also been identified. In the heart, HDAC4 represses the MEF2/MyoD regulated transcription of myogenic factors. miR-1 is induced by MEF2/MyoD and silences HDAC4, the resulting decrease in HDAC4 allows increased activity of MEF2/MyoD and thus mediates further expression of miR-1 loci (Fig. 2C) [82]. It is speculated that feed-forward circuits ensure sustained, robust expression of miRNAs and may play a role in promoting tissue-specific miRNA expression.

In addition to the stable changes in miRNA transcription modulated by developmental programs, miRNA can also be rapidly, and in some cases, transiently induced by growth factor signaling. Vascular smooth muscle cells (VSMC) retain the unique ability to undergo phenotypic switching between a differentiated 'contractile' state and a de-differentiated 'synthetic' state. This process is critically important for the repair of vessels following injury, but deregulation of phenotype switching contributes to vascular disorders, including hypertension, balloon-angioplasty-induced restenosis, and atherosclerosis. A critical player in the dedifferentiation of VSMC are the platelet derived growth factors (PDGFs), which promote reduced contractile gene expression, increased migration and increased proliferation of smooth muscle cells [83]. PDGF signaling results in a rapid, transcriptional induction of miR-221 which is required for multiple facets of the synthetic VSM response [58]. Interestingly, miR-222 which is a part of the miR-221 gene cluster, is not co-regulated by PDGF [58]. Induction of miR-221 promotes increased proliferation and migration as well as decreased contractile gene expression through distinct targets, indicating that a single miRNA can extrapolate an extracellular input to mediate multiple biological outcomes [58]. Other examples of growth factor-mediated activation of miRNA genes include miR-132 which is induced in neurons by brain derived neurotrophic factor (BDNF) and miR-192 which is induced in the diabetic kidney by transforming growth factor-β (TGF-β) [84,85]. Rapid induction of miRNAs in response to growth factors may serve to promote a switch in gene expression and are likely to be important for cellular responses upon cytokine stimulation.

## Epigenetic control of miRNAs

As the majority of miRNA genes are transcribed by the same RNA polymerase as protein coding genes, the mechanisms of epigenetic control known for protein coding genes are likely to also apply to miRNA loci. Aberrant DNA methylation of tumor suppressor genes is a common occurrence in cancer; accordingly, several miRNA loci including miR-9-1, -193a, -137, -342, -203 and -34b/c, are found to be hypermethylated in multiple human cancers [86,87]. Conversely, the let-7a-3 locus was found to be hypomethylated in lung adenocarcinoma and elevated expression of this locus resulted in enhanced oncogenic gene transcription [88]. miRNA promoters are also regulated by histone modifications during development and pathogenesis. Histone deacetylase (HDAC) inhibitors have been reported to up-regulate a subset of miRNAs, including miR-1 in cancer cells [89,90]. Alternatively, in the breast cancer cell line SKBr3, 32 mature miRNAs were significantly downregulated by treatment with HDAC inhibitors [91]. Interestingly, miR-27a was strongly repressed by HDAC inhibitor and was found to target genes previously shown to be up-regulated by HDAC treatment [91]. These findings suggest that changes in gene expression previously linked to HDAC activity may be partly due to altered miRNA expression. In normal development, acetylation of lysine 56 of histone H3 marks many genes responsible for embryonic stem cell pluripotency, including *nanog, sox2, oct4 *as well as 13 miRNAs highly expressed In stem cells [92]. In addition to transient regulation mediated by acetylation and methylation, miRNA are also subject to stable epigenetic control through genomic imprinting. Two well-characterized imprinted regions, H19 and the Dlk1-Gtl2 domains have recently been found to contain miRNA clusters [93,94]. Given that imprinted regions are often associated with human genetic disorders and differential imprinting is required for normal development, these miRNAs may have a yet unidentified regulatory role during development [95]. As in the case of protein coding genes, epigenetic modifications of miRNA loci provides an additional layer of transcriptional control in normal development and disease.

## The core Drosha processing machinery

Analysis of mature and primary transcripts of miRNAs in 68 tumor and 22 normal samples indicated that the correlation between the primary transcript and mature miRNA was disrupted in the tumor samples, perhaps suggesting global alterations in miRNA processing [96]. Furthermore, the levels of mature miRNA are generally decreased in tumors in comparison with normal tissues [33]. Experimental knockdown of miRNA processing factors, such as DiGeorge syndrome critical region 8 (DGCR8), Drosha, or Dicer in mouse lung adenocarcinoma cells dramatically elevates properties of tumorigenesis, including proliferation, soft agar colony formation, and *in vivo *tumor burden [97]. Together, these results indicate that disrupted miRNA biogenesis can alter the delicate balance of normal cellular physiology.

Following transcription, the primary miRNA undergoes two cleavage steps to generate the mature miRNA (Fig. 1). The first cleavage is catalyzed in the nucleus by the RNase III enzyme Drosha [40,42,98]. Pri-miRNAs generated by the transcriptional machinery are generally several thousands of nucleotides long and contain a distinctive stem loop structure. Drosha cleaves at the base of the stem to generate a ~60–100 nt hairpin RNA with a characteristic 2 nt 3' overhang (Fig. 1) [40]. The Drosha processing of a subset of miRNAs can be closely coupled with pri-miRNA transcription. Co-transcriptional Drosha processing occurs for independently transcribed (intergenic) miRNAs as well as those embedded within the introns of host genes [51]. Nuclear fractionation studies indicate that some pri-miRNAs may remain associated with chromatin following transcription [51,53]. Furthermore, mutation of the pri-miRNA cleavage and polyadenylation signal enhances association of the pri-miRNA with chromatin and increases the efficiency of Drosha cleavage [53]. It is well established that many pre-mRNA processing activities, such as 5' capping, splicing, and 3' poly(A) adenylation, occur while pre-mRNAs are undergoing transcription. Therefore it is perhaps not too surprising that Drosha cleavage of some pri-miRNAs also occurs co-transcriptionally. The association of Drosha with nascent transcripts may enable further control of processing though the recruitment of additional factors or altered rates of the elongation step of transcription mediated by RNA pol II.

The precise position and orientation of Drosha cleavage is critically important for the generation of mature miRNA because the second cleavage step occurs at a defined distance from the free end generated by Drosha. Therefore, Drosha cleavage determines the identity of both the 5' and 3' nt of the mature miRNA. The slightest error in Drosha cleavage could alter not only the seed sequence, but could also invert the relative stability of the two strands, resulting in the incorporation of the improper miRNA strand into the RISC complex. Recombinant Drosha can promote RNA cleavage *in vitro*, however, the position of cleavage is imprecise [41]. *In vivo*, Drosha is present in a large protein complex (minimally 650 kDa in human cells) and the association of Drosha with co-factors present within this complex promotes the fidelity and activity of Drosha cleavage. In particular, DGCR8 is required for Drosha-mediated cleavage of pri-miRNAs [99,100]. The pri-miRNA is composed of a ~33 nt stem connected by a terminal loop and flanked by single-stranded segments. DGCR8 is thought to recognize the region between the single-stranded RNA and the stem in order to direct Drosha cleavage one helical (~11 bp) turn away from this junction [101]. Some miRNAs contain large terminal loops that may be structurally similar to the single-stranded flanking RNA [98]. *In vitro*, the recognition of a terminal loop by the DGCR8-Drosha complex could result in cleavage at a site ~11 bp away from the loop-stem junction [101]. This placement of Drosha results in an abortive cleavage within the mature miRNA sequence and leads to the reduction of mature miRNA expression. To prevent abortive processing *in vivo*, the correct positioning of Drosha on the hairpin structure may be facilitated by additional co-factors. Furthermore, while the cropping of many miRNAs can be mediated *in vitro *by purified DGCR8 and Drosha, nuclear run-on and *in vitro *processing assays indicate that pri- to pre- cleavage of some miRNA is relatively slow and inefficient [51]. Therefore, the efficient processing of some miRNA by the Drosha/DGCR8 complex may require the involvement of accessory factors.

Drosha interacts with a wide variety of proteins in addition to DGCR8; mass-spec analysis indicates that flag-tagged Drosha expressed in HEK293 cells is associated with at least 20 distinct polypeptides [41]. These associated proteins may be required for the Drosha processing of specific miRNA sequences/structures or involved in the differential regulation of processing through 1) prevention of abortive processing, 2) alteration of the miRNA hairpin structure, 3) enhanced recruitment or positioning of Drosha on the hairpin, or 4) increased RNA cleavage activity. As discussed below, the biogenesis of specific groups of miRNAs are altered by Drosha binding partners which interact in a sequence- or structure-specific manner with pri-miRNAs. These mechanisms provide an important method for the control of miRNA expression during development, and deregulation of these processes is likely to contribute to multiple pathological outcomes.

## Modulation of Drosha activity

### The DEAD-box RNA helicases p68/p72

The DEAD-box RNA helicases p68 (DDX5) and p72 (DDX17) were identified as components of the large Drosha processing complex by immunoprecipitation-mass spectrometry analysis and subsequently shown to also associate with DGCR8 [41,102]. Knockout of either p68 or p72 in mice is lethal (E11.5 or P2, respectively) and double knockout animals show earlier lethality without obvious specific degeneration of organogenesis [103]. Analysis of mature miRNA levels by microarray indicated reduced steady state levels of ~35% (94 out of 266 surveyed) miRNA in p72 null MEFs vs. control. Most of the miRNAs which were reduced by p72 knockout were similarly reduced by p68 knockout, and combined reduction of p68 and p72 did not further reduce mature miRNA levels; suggesting that the two proteins are largely redundant or function as a heterodimer. Interestingly, while the level of pre-miRNA was also reduced in p72 or p68 knockout cells, the level of pri-miRNA was unchanged. Furthermore, *in vitro *processing assays showed attenuation of Drosha processing from extracts depleted of p68/p72. *In vivo *RNA-immunoprecipitation (RNA-IP) studies indicated reduced Drosha binding to pri-miR-199a in the absence of p68/p72. Together, these results strongly support a role for p68/p72 in promoting the Drosha processing of a sub-set of miRNAs [103]. The precise mechanism of p68/p72-mediated processing is unclear, but may involve rearrangement of the RNA hairpin which results in enhanced Drosha recruitment or stability. Alternatively, as p68/p72 are known to interact with a variety of proteins, including RNA processing enzymes and transcription factors, they may serve as a scaffold for the recruitment of multiple protein factors to the Drosha microprocessor [104,105].

### The Smads

**Figure 4:**
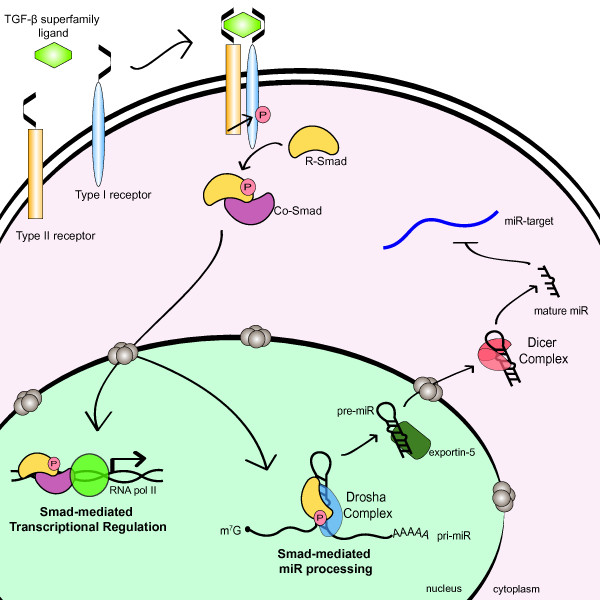
**Regulation of miRNA maturation by the TGF-β superfamily signaling**. TGF-β and BMP signaling stimulates the production of pre-miR-21 by promoting the Drosha-mediated processing by controlling nuclear localization of R-Smad proteins. Thus, Smads regulate gene expression in two distinct manners; (i) transcriptional regulation by DNA binding and (ii) regulation of miRNA maturation by associating with the Drosha/DGCR8 complex.

The role of p68/p72 in miRNA processing is further supported by the positive regulation of Drosha processing mediated by the p68-interacting Smad proteins. The Smads are the signal transducers of the Transforming Growth Factor-β (TGF-β) family signaling cascade. In the canonical pathway, ligand binding to the type I and type II TGF-β receptors promotes the nuclear accumulation of receptor-Smads (R-Smads) in association with the common-Smad (co-Smad), Smad4 (Fig. 4). The complex of R-Smad and co-Smad bind to sequence elements within the promoter of target genes to positively or negatively regulate gene transcription. TGF-β and its family member, Bone Morphogenetic Protein 4 (BMP4) are particularly important for the differentiation of VSMCs. Treatment with either BMP4 or TGF-β increases expression of contractile smooth muscle genes; this process is due, at least in part, to the miR-21-mediated repression of programmed cell death protein-4 (PDCD4). miR-21 is rapidly induced by BMP4 and TGF-β in VSMC which results in a subsequent decrease in PDCD4 and increased smooth muscle gene expression [106]. Interestingly, although knockdown of the R-Smads prevented upregulation of mature and pre-miR-21 in response to BMP4, no alteration in pri-miR-21 transcription could be detected. Furthermore, BMP4 could increase the expression of pre- and mature miR-21 driven by a heterologous promoter, suggesting that miR-21 is post-transcriptionally regulated at the Drosha processing step by the Smads. The identification of R-Smads as binding partners of p68 by yeast-two-hybrid suggested that R-Smads could be associated with the Drosha complex [107]. Consistently, co-immunoprecipitation and RNA-ChIP studies indicate Smad is present in a complex with Drosha and p68 on the pri-miR-21 hairpin following BMP4 or TGF-β stimulation (Fig. 4) [106]. An increase in Drosha binding to pri-miR-21 was also observed following ligand treatment, suggesting that Smads may promote the association of Drosha with miRNA hairpins. These results indicate that in addition to transcriptional regulation mediated by the Smads, TGF-β can regulate gene expression through miRNA processing (Fig. 4). A distinguishing feature of the two pathways is the requirement for the co-Smad4. While Smad4 is required for transcriptional responses to TGF-β, the association of R-Smads with the Drosha processing machinery does not require Smad4. Knockdown of Smad4 in VSMC did not affect induction of miR-21; furthermore, miR-21 is strongly induced by TGF-β in the Smad4-null MDA-MB-468 breast cancer cell line [106]. Several other miRNAs are post-transcriptionally induced by BMP and TGF-β, suggesting that rapid modulation of miRNA levels may play an important role in cellular response to cytokine signaling (B. Davis and A. Hata, unpublished observation) [106]. Intriguingly, knockdown of Smad nuclear interacting protein 1 (SNIP1) in HeLa cells reduces the expression of several mature miRNAs, including miR-21 [108]. Furthermore, SNIP1 is found in complex with RNA processing enzymes [109] as well as Drosha [108]. The Arabidopsis homologue of SNIP1, DAWDLE, is required for efficient pri- to pre-miRNA processing and is thought to promote the access or recognition of pri-miRNA by DCL1 [108]. These results suggest that Smads may be present in large, multi-protein complexes which regulate mature miRNA levels through alteration of miRNA processing in a context dependent manner.

### p53

The tumor suppressor protein p53 was recently identified to modulate miRNA processing through association with p68 and Drosha, similar to the Smads. Under conditions of DNA damage, several miRNAs, including miR-143 and miR-16, are post-transcriptionally up-regulated [110]. p53 is required for this process as p53-null HCT116 cells do not increase miRNA in response to DNA damage. Co-immunoprecipitation studies indicate that p53 is present in a complex with both Drosha and p68, and addition of p53 to *in vitro *processing assays could enhance Drosha processing [110]. Interestingly, several p53 mutants that have been previously linked to oncogenic progression suppressed miRNA expression [110]. These results indicate that the association of p68/Drosha with accessory factors, such as p53 or Smads, may be particularly important for the rapid induction of miRNAs in response to extracellular stimuli. It intriguing to speculate that other p68-interacting proteins, such as estrogen receptors, MyoD, and Runx2 [111-113], might also have a role in the regulation of miRNA processing.

### The heterogeneous nuclear ribonucleoprotein A1 (hnRNP A1)

An additional example of miRNA specific processing regulation is mediated by hnRNP A1 which is required for the processing of a member of the miR-17-92 cluster, miR-18a. Addition of hnRNP A1 to *in vitro *processing assays dramatically increases the conversion of pri-miR-18a to pre-miR-18a. Conversely, depletion of hnRNP A1 lowers pre-miR-18a levels, but does not alter the expression of the other 5 clustered miRNAs [60]. Further studies indicate that direct binding of hnRNP A1 to the loop region of miRNA hairpins causes the structural rearrangement of the hairpin to generate a more favorable Drosha cleavage site. The addition of 2'O-methyl oligonucleotides ('looptomiRs') complementary to the loop region inhibits hnRNP A1 binding and prevents hnRNP A1-mediated Drosha processing. The loop region of miR-18a is well conserved throughout evolution, emphasizing the importance of the hnRNP A1-loop interaction for the processing of miRNA. Interestingly, approximately 14% of human miRNAs contain highly conserved loop regions, suggesting that processing regulation by hnRNPs may extend well beyond miR-18a. Consistently, the processing of two other miRNAs bearing conserved loops, miR-101 and let-7a-1 is also prevented by complementary looptomiRs (Fig. 3) [114]. The human genome encodes at least 19 hnRNPs, many of which have distinct RNA binding properties and tissue distribution [115,116]. Indeed, miR-101 and let-7a-1 were found to associate with hnRNP L and hnRNP I, respectively [114]. Although the functional requirement for the association of other hnRNPs is unknown, it suggests that the large family of hnRNPs could serve as accessory factors in the regulated processing of a variety of miRNAs. The differential expression of hnRNP proteins as well as their sub-cellular localization may serve to regulate miRNAs in a tissue or context-dependent manner. For example, phosphorylation of hnRNP A1 by p38 MAPK has been reported to promote the cytoplasmic localization of hnRNP A1 [117,118]. These results suggest that, similarly to smads, hnRNP A1 may control miRNA processing in response to extracellular stimuli.

### The KH-type splicing regulatory protein (KSRP)

The importance of the loop region in the regulation of miRNA processing is further supported by recent studies which indicate KSRP directly interacts with G-rich regions present within the loop of a sub-set of miRNAs to promote both Drosha and Dicer mediated miRNA processing [119]. Transient knockdown of KSRP in HeLa cells strongly reduced the expression of a group of mature miRNAs, including let-7a, and miR-206 (Fig. 3). Knockdown of KSRP altered cellular responses mediated by these miRNAs, including proliferation and skeletal muscle differentiation. KSRP was found to be present in both the Drosha and Dicer complexes, and knockdown of KSRP reduced the processing activity of Drosha and Dicer [119]. The interaction of KSRP with sequential miRNA processing steps may serve to promote the synergistic activation of processing for a subset of miRNA. Alternatively, KSRP association with either the Drosha or Dicer complexes may be sufficient to alter the miRNA processing in response to cellular stimuli. For example, lipopolysaccharide (LPS) treatment of macrophages dramatically increases mature miR-155, without significantly altering expression of the primary transcript. Mobility shift assays indicate KSRP interacts with pri-miR-155, and knockdown of KSRP prevents LPS-mediated increase of miR-155 [120]. Together these results suggest a role for KSRP in promoting the post-transcriptional regulation of miR-155 in response to LPS. Although the mechanism of this regulation is unknown, it is intriguing to speculate that the sub-cellular localization or modification of KSRP could be regulated by LPS. Indeed, phosphorylation of KSRP Serine 193 by Akt promotes nuclear accumulation of KSRP through interaction with 14-3-3ζ [121]. An important future challenge is the determination of the signaling pathways which modulate the activity of miRNA processing accessory factors such as KSRP and hnRNPs.

### Lin-28

In addition to the positive regulation of miRNA processing discussed above, negative regulation of the Drosha processing of some miRNAs has also been reported. The let-7 family of miRNAs is often present as multiple copies in the genome; the human genome contains 9 mature let-7 sequences derived from 12 precursors [122]. While most let-7 primary transcripts are highly expressed throughout development, the mature let-7 is detectable only in highly differentiated cells, suggesting a mechanism of post-transcriptional regulation [122]. Analysis of the primary miRNA sequences of the human let-7 family as well as let-7 from divergent species indicated the presence of conserved nucleotide sequences present in the hairpin loop, suggesting a possible regulatory role for this region [123,124]. While pri-let-7 is processed efficiently *in vitro *using cell extracts derived from differentiated cells, processing is reduced in extracts derived from undifferentiated cells. The addition of short oligonucleotides corresponding to the loop region to *in vitro *processing reactions prevents inhibition of Drosha processing in cell extracts, presumably by competing for inhibitor binding [123]. Affinity purification and mass-spec identified Lin-28 as the let-7 interacting protein responsible for processing inhibition [123,125]. Furthermore, crosslinking, followed by electrophoretic mobility shift assay (EMSA) confirmed that the loop region interacts with Lin-28, and disruption of the Lin-28 binding site within the loop prevented Lin-28-mediated repression of let-7 (Fig. 3) [123,124]. Further support for the physiological relevance of let-7 regulation by Lin-28 comes from the observation that Lin-28 and mature let-7 show reciprocal expression patterns during development.

The precise mechanism of Lin-28 inhibition of Drosha processing is unclear. Lin-28 has a strong affinity for let-7, perhaps suggesting Lin-28 could prevent interaction with Drosha/DGCR8 by simple competition. Alternatively, analogous to hnRNP A1 discussed above, Lin-28 interaction with the loop region could rearrange the secondary structure of the hairpin and, in this case, discourage Drosha processing. It is interesting to note that Lin-28, KSRP, and hnRNPs all interact with conserved nucleotides present within the loop of pri-miRNAs. The balanced interaction of proteins with the miRNA loop may allow for the fine-tuning of miRNA levels during development or in response to extracellular cues. For example, although KSRP interacts strongly with pri-let-7g in differentiated cells, high levels of Lin28 block KSRP association in undifferentiated p19 cells [119].

### The nuclear factor 90/45 (NF90/NF45)

Repression of Drosha processing of let-7 family members is also mediated by NF90 and NF45 proteins (Fig. 3) [126]. *In vitro *and *in vivo *processing assays indicate that the NF90/NF45 complex reduces pre-miRNA while increasing pri-miRNA levels. The NF90/NF45 family of proteins interacts with small double-stranded RNAs, including the adenovirus VA RNA [127] consistently, the NF90/NF45 complex binds pri-miRNAs with high affinity. EMSA analysis suggests that the high affinity association of NF90/NF45 complex with pri-miRNA precludes interaction with DGCR8, thus reducing production of pre- and mature miRNA [126]. Unlike Lin-28, which specifically regulates let-7, the NF90/NF45 complex may be more promiscuous, as the processing of 3 other analyzed miRNAs is also modulated.

Altered Drosha processing has been described for the tumor suppressor miR-7 in glioblast4oma [128]. While the primary miR-7 transcript is expressed at equal levels in normal and tumor samples, the precursor and mature miRNA is dramatically reduced in glioblastoma tumor samples and cell lines. Restoration of miR-7 levels to primary cell lines reduced the viability and invasiveness by targeting the EGF Receptor and inhibiting the Akt signaling cascade [128]. As the levels of several other miRNA were unaffected, the deregulation of processing seems specific to miR-7. Although the mechanism is unknown, reduced pre-miR-7 in the tumor could arise from either (i) increased expression of a processing inhibitor or (ii) decreased expression of a processing activator. It will be interesting to determine if miR-7 is regulated by one of the mechanisms described above, or if yet-unidentified mechanisms are responsible for the altered regulation of miR-7 in cancer cells.

### Regulation of Drosha/DGCR8 expression

Finally, it has been reported that the total levels of Drosha and DGCR8 in the cell are tightly controlled and may play a role in the regulation of miRNA processing. Increased Drosha expression was identified in late stage cervical cancer samples and was associated with poor prognosis of esophageal cancer patients [129,130]. Interestingly, despite a 2–7 fold increase in Drosha expression in cervical cancer samples, only miR-31 was increased, while the other differentially expressed miRNAs were decreased [130]. DGCR8 was originally identified as a gene located within a common chromosomal deletion at ch22q11, which gives rise to DiGeorge syndrome, including learning disabilities and heart defects [131]. Mouse models of this chromosomal deletion exhibit a moderate decrease of 59 mature miRNAs [132]. Given the critical role of Drosha and DGCR8 in mediating miRNA processing, it is surprising that relatively minor changes have been found in miRNA levels upon dramatic alteration of DGCR8/Drosha levels. These findings may be explained, at least in part, by the recent finding that the total levels of Drosha and DGCR8 are coupled in an intricate feed-back circuit. In addition to cleaving pre-miRNA hairpins, the microprocessor complex can also promote cleavage of hairpin structures within annotated protein coding genes which are not further processed by Dicer [133,134]. This type of cleavage allows Drosha to modulate protein coding gene expression independent of miRNA production. Indeed, tiling microarray analysis in drosophila S2 cells following Drosha depletion indicates that many annotated protein coding genes, including *DGCR8*, are targeted for degradation through this pathway [133]. Therefore, Drosha maintains a highly regulated level of DGCR8 through the microprocessor-mediated cleavage of DGCR8 mRNA. Additionally, DGCR8 stabilizes Drosha protein levels and ensures the tight coupling of the core microprocessor proteins. This regulatory loop seems to be highly effective *in vivo *as in the DGCR8 heterozygous knockout mouse the protein levels of DGCR8 are only modestly reduced despite a 50% decrease in genomic DGCR8 [134]. The presence of this mechanism suggests that the relative levels of DGCR8 and Drosha must be closely coupled for accurate miRNA processing. Interestingly, *in vitro *processing experiments using recombinant proteins indicate that as little as a 3-fold excess of DGCR8 over Drosha dramatically inhibits processing activity of Drosha [41]. In addition to alteration of protein levels, the overall activity of the Drosha/DGCR8 complex may be altered under some circumstances. Cells grown to high density exhibit increased pre- and mature miRNA expression without alteration of Drosha or DGCR8 protein level. Furthermore, extracts from high-density cells promoted the more efficient conversion of pri- to pre-miRNA in *in vitro *processing assays [135]. Although the mechanism of this increased processing is unknown, it is possible that post-transcriptional modifications or association with accessory factors could alter the activity of Drosha or DGCR8. For example, the binding of heme to DGCR8 promotes dimerization of DGCR8 and facilitates pri-miRNA processing [136].

## Drosha-independent miRNA maturation: mirtrons

A sub-set of miRNAs which by-pass the Drosha cleavage step, called 'mirtrons', have been identified. These miRNAs are embedded within short introns and the ends of the pre-miRNA hairpin are determined by the 5' and 3' splice sites of the intron [137,138]. Following the completion of splicing, the lariat-debranching enzyme resolves the branch point to generate a pre-miRNA-like hairpin that can be exported from the nucleus and further processed by Dicer. While fly and worm mirtrons generate hairpins with 2-nt 3' overhangs consistent with the structure of canonical miRNAs, mammalian mirtrons generally contain single nucleotide overhangs on both strands [139]. In general, mirtrons compose only a small percentage of genomically-encoded miRNAs and are usually expressed at a lower level than canonically processed miRNAs. While fly, worm, and mammals utilize a similar mechanism for the generation of miRNAs in the absence of Drosha activity, the sequences of mirtrons are not evolutionarily conserved. Furthermore, as most mirtrons have appeared recently on an evolutionary time scale, it is intriguing to speculate that the conversion of a short intron into a mirtron may serve as a simple mechanism for the creation of new regulatory RNAs during evolution [139].

## miRNA export by Exportin-5

The pre-miRNA hairpin is translocated from the nucleus into the cytoplasm through interaction with exportin-5 (Fig. 1) [140]. Like other nuclear export receptors, exportin-5 cooperates with the small GTPase Ran to mediate directional transport of proteins. Pre-miRNAs interact with exportin-5 and the GTP-bound form of Ran in the nucleus. Following export to the cytoplasm, GTP is hydrolyzed and the pre-miRNA is released from exportin-5 [43,141]. Interaction of exportin-5 with RNA is independent of sequence, but requires a double-stranded region of at least 16 bp [43,142]. Additionally, a 3' overhang facilitates interaction of the pre-miRNA with exportin-5, while a 5' overhang is inhibitory for the association [142]. Exportin-5 may be particularly well suited to recognize and export correctly processed miRNA precursors since Drosha catalyzed cleavage of pri-miRNAs generates a 2-nt 3' overhang.

Knockdown of exportin-5 decreases the levels of cytoplasmic miRNA and reduces the ability of both exogenous and endogenous miRNAs to target a luciferase sensor construct. No accumulation of pre-miRNAs in the nucleus was observed in the absence of exportin-5, suggesting that exportin-5 may also serve to stabilize pre-miRNAs [140]. The level of exportin-5 in the cell may be rather limiting as either overexpression of pre-miRNA or addition of an alternative exportin-5 substrate, adenovirus non-coding RNA VA1, blocks nuclear export of radio-labeled pre-miRNA [141,143]. Furthermore, saturation of the exportin-5 machinery is known to contribute to the toxicity in liver resulting from high expression of shRNA in mouse [144]. Conversely, overexpression of exportin-5 could enhance the activity and total level of exogenous and endogenous miRNAs [145]. These results are consistent with nuclear export representing a rate-limiting step in miRNA biogenesis.

The export of pre-miRNA from the nucleus to the cytoplasm may be differentially regulated under certain conditions. For example, the precursor hairpins of miR-105, -128, and -31 are detected in many cells at relatively high abundance. However, under some conditions the mature miRNA is undetectable [26]. Specifically, northern blot and real time PCR indicate that pre-miR-31 is expressed at comparable levels in the pancreatic cancer cell line HS766T and the MCF7 breast cancer cell line. However, while HS766T cells show high levels of mature miR-31, no expression is detected in the MCF7 cells. *In situ *hybridization for pre-miR-31 showed the expected cytoplasmic localization in HS766T cells, while in MCF7 cells pre-miR-31 accumulated in the nucleolus [26]. Together, these results indicate that the export of pre-miR-31 is regulated in a cell-type dependent manner; however, the mechanism of altered miRNA export is unknown. The precise sequence of the miRNA hairpin alters the efficiency of exportin-5 mediated export in *Xenopus *oocyte extracts, suggesting that RNA secondary structure may lead to differential miRNA export [141]. Alternatively, because the altered export of miR-31 is observed in a context-dependent manner, an unknown RNA binding factor may be involved in regulating the export of some miRNAs.

## Control of Dicer cleavage and RISC loading

### Dicer Cleavage and Accessory factors

Following translocation into the cytoplasm, the pre-miRNA is cleaved near the terminal loop by the RNase type III protein Dicer to generate a ~22-nt double-stranded miRNA [146,147] (Fig. 1). Like Drosha, Dicer generates 2-nt 3' overhangs upon cleavage. Dicer is highly conserved throughout evolution and present in nearly all eukaryotic organisms; the mammalian Dicer is composed of multiple domains including the DExD/H-box helicase, DUF283, dsRBD, PAZ and ribonuclease domains [148]. Structural analysis of the *Giardia intestinalis *Dicer, composed of the PAZ and ribonuclease domains, indicates that the PAZ domain binds the 3' overhangs at the end of double-strand RNA to position the RNA for cleavage by the two catalytic RNase III domains [149]. Interestingly, replacement of the PAZ domain with a U1A RNA binding domain resulted in altered end-recognition but maintained the length of generated miRNAs [149]. These findings indicate that Dicer serves as a molecular ruler to cleave miRNA at a specific distance from the ends generated by Drosha [150]. The *Giardia *Dicer likely represents a minimal functional Dicer protein and the additional domains present in mammalian Dicer may serve to allow more complex regulation of Dicer activity. Indeed, analysis of the processing ability of Dicer mutants indicates that the helicase domain may be especially critical for the processing of hairpins containing thermodynamically unstable regions [151].

Dicer is associated with the double-stranded RNA binding proteins TAR RNA binding protein (TRBP) and protein kinase R-activating protein (PACT) [152,153]. Depletion of either of these cofactors decreases the steady-state levels of Dicer protein [153,154]. Furthermore, truncation mutations of TRBP are found in carcinoma and associated with both decreased miRNA levels, as well as dramatic destabilization of Dicer [155]. Dicer protein levels can be rescued by wild type TRBP, suggesting that the interaction between Dicer and TRBP is a critical determinant of Dicer stability [155]. Additionally, the RNA binding protein fragile × mental retardation protein (FMRP) is associated with multiple components of the miRNA pathway, including Dicer [156]. Interestingly, the phosphorylation of FMRP prevents association with Dicer, but promotes interaction of FMRP with pre-miRNA [157]. Although the consequences of FMRP phosphorylation on microRNA processing are unclear, these results suggest a mechanism for the regulation of Dicer activity through the phosphorylation of associated proteins.

### The RISC Complex

Although recombinant Dicer can catalyze the efficient and specific cleavage of RNA *in vitro*, these miRNAs are inefficiently incorporated into the RISC complex [45]. Furthermore, an exogenous pre-miRNA hairpin is more effective at promoting the silencing of targets than a duplex RNA, suggesting that the coupling of Dicer processing and RISC loading is critical for the proper function of miRNAs [45]. The primary component of the RISC complex, and the effectors of miRNA-mediated repression are the Argonaute (Ago) proteins. The human genome contains eight Ago-family proteins (Ago1-4 and Piwi1-4) [158]. Although structurally similar to Agos, the Piwi family members interact with piRNAs and function in germline maintenance; reviewed in [159]. While all of the Ago proteins have the ability to interact with small RNAs, Ago2 is the only one with RNA cleavage activity and is thought to play a prominent role in miRNA-mediated silencing.

*In vivo*, Ago2 associates with Dicer and TRBP/PACT to form the RISC Loading Complex (RLC) which allows the tight coupling of Dicer cleavage to the incorporation of miRNA into the RISC complex [153]. *In vitro *reconstituted RLC composed of recombinant Dicer, TRBP, and Ago2 efficiently catalyzes pre-miRNA cleavage in an ATP-independent manner [160,161]. Cleavage by Dicer results in an unstable double-stranded RNA composed of the active guide strand (miRNA) and the passenger (miRNA*) strand (Fig. 1). An important role of the RLC is the unwinding of the double-stranded miRNA, which is followed by incorporation of the guide strand into the miRNA-containing ribonucleoprotein (miRNP) complex and degradation of the passenger strand. The mechanism of strand selection in humans is unclear, however, the guide strand generally exhibits lower thermodynamic stability of the 5' end [162]. Deep sequencing of small RNAs indicates that in most cases, the ratio of miRNA:miRNA* strand is ~100:1, suggesting a possible mechanism of active degradation of miRNA* but not miRNA [163]. Although driven by thermodynamic properties, the assembly of RNA into the RISC complex may be subject to additional regulation as the ratio of miRNA:miRNA* can vary dramatically depending on the specific properties of the miRNA duplex. Furthermore, the ratios of miRNA:miRNA* are altered in different tissues and developmental stages [164,165]. These findings suggest that differential strand selection could represent a yet unappreciated mechanism of miRNA regulation. Recombinant RLC can mediate strand selection of pre-let-7a, indicating that this process is intrinsic to the Dicer-TRBP-Ago2 complex, but may be modified by additional factors in a context dependent manner [161].

The presence of a pre-formed Ago2-Dicer complex may be particularly important for miRNAs containing a high degree of complementarity along the hairpin. These miRNAs are cleaved by Ago2 in the middle of the presumptive passenger strand prior to Dicer processing [166]. This nicking may facilitate strand selection and dsRNA unwinding of some miRNAs. However, as Ago2 is the only Ago family member with detectable endonuclease activity, strand-selection must also occur without cleavage of the passenger strand [167,168]. Following the loading of Ago with the single-stranded guide miRNA, Dicer dissociates and the effector complex (miRNP) capable of selecting miRNA targets is formed through the association of additional proteins [160,169].

### Regulation of Dicer Activity

The relatively low levels of pre-miRNA relative to pri-miRNA or mature miRNA suggest that Dicer is generally very efficient at processing pre-miRNA [26,170]. In comparison to Drosha, which often requires the addition of accessory factors to promote processing, regulation of miRNAs at the Dicer step often involves inhibition of Dicer activity. Early evidence for post-transcriptional control of miRNA at the level of Dicer processing comes from studies of miRNA expression in colorectal neoplasia. Mature miR-143 and miR-145 are expressed at much lower levels in tumor samples than normal tissue. However, northern blots indicate that the level of pre-miR-143 or -145 is not significantly different in the tumor samples, suggesting altered dicer processing in the colorectal tumor samples [171]. Mature miR-143 and miR-145 are also decreased in several cancer cell lines as well as in B-cell malignancies [171,172]. These miRNAs act as tumor suppressors through the repression of the mitogen activated protein kinase ERK5 [173]. Although the mechanism of processing control is unknown, these results suggest that restoration of normal miRNA processing may represent a novel therapeutic option for the treatment of some tumors.

miRNA processing by Dicer is also differentially regulated during normal development and tissue specification. For example, while pre-miR-138 is ubiquitously expressed in all tissues and HeLa cells, the mature miR-138 is only detected in adult mouse brain and fetal liver [174]. An alteration in nuclear export can be ruled out because the precursor is effectively exported into the cytoplasm, suggesting regulation of Dicer processing. Furthermore, while pre-miR-138 is processed efficiently by Dicer *in vitro*, the addition of HeLa cell extract inhibits the processing of pre-miR-138. HeLa extract does not alter the processing of other miRNAs normally expressed in HeLa cells [174]. These results suggest that HeLa cells, and most tissues, express a yet-unknown factor which specifically inhibits Dicer activity on miR-138.

In addition to inhibition of the Drosha processing step discussed above, Lin-28 also inhibits the Dicer processing of let-7 family members (Fig. 3) [125,175]. Lin-28 shuttles between the nucleus and cytoplasm, but is commonly enriched within the cytoplasm, suggesting that the cytoplasm may be the primary location of the Lin-28:let-7 interaction. Addition of purified Lin-28 decreased the association of let-7 with Dicer, and reduced Dicer-mediated *in vitro *processing, suggesting that Lin-28 can compete with Dicer for access to let-7 [125]. An additional cytoplasmic blockade of let-7 processing by Lin-28 has recently been described by Heo and colleagues (Fig. 3) [176]. In hepatocellular carcinoma cell lines, Lin-28 promotes the 3'-uridylation of pre-let-7 which inhibits Dicer processing and promotes degradation of pre-let-7. This activity requires the zinc-finger domains of Lin-28, as mutations in this region maintain interaction with let-7 but do not induce uridylation [176]. The identity of the uridylating enzyme recruited by Lin-28 awaits further identification. Although Lin-28 promotes the uridylation of only let-7 family members, it will be interesting to determine if other pre-miRNAs are similarly regulated by the addition of oligonucleotides.

### Regulation of Dicer expression

The total levels of Dicer may serve as an important control point in miRNA biogenesis. A careful analysis of the Dicer 5'UTR indentified multiple alternatively spliced leader exons that are expressed in a tissue specific manner [177]. Although differential splicing alters the translation efficiency of *dicer in vitro*, the biological relevance of alternatively spliced *dicer *isoforms is unknown. Altered expression of Dicer is observed in several types of human cancer, for example, Dicer is increased in prostate tumors, as well as in a Burkitt's lymphoma derived cell line [178-180]. Conversely, Dicer expression is decreased in non-small-cell-lung cancer, and reduced Dicer correlates with poorer prognosis [181]. These conflicting trends may be indicative of cell type differences or tumor stage. Analysis of precursor lesions of lung adenocarcinoma showed increased Dicer expression, while advanced invasive adenocarcinoma showed decreased protein levels [182]. The importance of controlled Dicer levels in normal physiology is further emphasized by the identification of let-7 target sites within *dicer *[8,183]. As the maturation of let-7 is dependent on Dicer, this interaction represents a negative feedback loop which may serve to regulate the total rate of miRNA biogenesis (Fig 3).

Dicer may also be regulated post-transcriptionally. The catalytic activity of Dicer processing of some miRNAs is increased by removal of the amino-terminal helicase domain, indicating that this domain may serve an autoinhibitory function [184]. In adult mouse neurons, the protease Calpain has been suggested to enhance Dicer activity, perhaps indicating that the proteolytic removal of the Dicer helicase domain could serve as a regulatory mechanism *in vivo *[185]. Finally, association with TRBP may activate Dicer by promoting the structural rearrangement of the Dicer helicase domain [184,186]. A more complete understanding of the regulation of Dicer activity is an important challenge for future studies.

### The role of Ago expression in miRNA regulation

The total levels of the Ago proteins within the cell also contribute to global miRNA regulation and biogenesis. Ectopic expression of Ago1-4 resulted in a dramatic increase of mature miRNAs [166]. In contrast, mouse embryonic fibroblasts and hematopoietic cells from Ago2 knockouts showed reduced levels of all mature miRNAs tested [166,187]. Some accumulation of pre-let-7 was observed in Ago2 knockout cells, suggesting that the presence of Ago2 in the RLC may enhance Dicer cleavage [166]. The dramatic increase in mature miRNA mediated by increased Ago expression could also indicate that Ago proteins are limiting and serve to stabilize miRNA. If this is the case, one might expect that Ago protein levels may be linked to the level of small RNA present within the cell. Indeed, a retrospective study of publically available microarray data from siRNA treated cells indicated an increase in Ago2 mRNA independent of the siRNA sequence [188]. Ago2 mRNA and protein expression was also found to be increased in a subset of breast cancer cells. Additionally, treatment with Epidermal Growth Factor (EGF) enhanced Ago2 stability, suggesting that alteration of Ago2 levels can occur at both the transcriptional and post-transcriptional level [189]. Recently, Ago proteins have been identified as substrates of the α-(P4H-α(I)) and β-(P4H-β) subunits of the type I collagen prolyl-4-hydroxylase (C-P4H) [190]. The hydroxylation of Ago2 at proline 700 enhanced Ago2 stability, while knockdown of the hydroxylating enzymes P4H-α(I) or P4H-β reduced Ago2 protein level [190]. The P4H-α(I) subunit is rate-limiting for the formation of active C-P4H and has been reported to be up-regulated by a variety of factors, including hypoxia and transforming growth factor-β. Given that these treatments each induce many miRNAs, one could hypothesize that concomitant up-regulation of Ago-2 by C-P4H hydroxylation may serve to stabilize the newly synthesized miRNAs.

## RNA editing

Adenosine deaminases that act on RNA (ADAR) enzymes catalyze the conversion of adenine to inosine (A to I) in double-stranded RNA. The resultant inosine has biochemical properties similar to guanosine and preferentially base pairs with cytosine, therefore altering the base pairing and RNA secondary structure of the edited RNA. Several studies have demonstrated that pri-miRNAs and pre-miRNAs are substrates of ADAR enzymes. The editing of miR-142 alters the secondary structure of the pri-miRNA and prevents cleavage by Drosha [191]. The edited, unprocessed pri-miR-142 is then rapidly degraded by the inosine-specific ribonuclease Tudor-SN [191]. miR-151 is also edited by ADARs; editing can occur on either the pri- or pre-miRNA species and prevents Dicer processing, resulting in the accumulation of pre-miRNA hairpins [192]. miR-151 editing occurs at high frequencies in a tissue-specific manner, suggesting that differential editing may underlie tissue-specific expression of some miRNAs [192]. An analysis of 209 pri-miRNA sequences identified 47 which are highly edited in the human brain and *in vitro *processing reactions indicated that while many edited pri-miRNAs were refractory to processing by Drosha or Dicer, the editing of selected miRNAs did not alter, or enhanced processing [193]. If miRNA processing is unaffected, the presence of edited nucleotides within mature miRNA could shift the target specificity. For example, a single A-to-I conversion within the seed sequence of miR-376 results in the regulation of PRPS1, an enzyme involved in purine metabolism, which is not targeted by the un-edited miRNA. ADAR2 null mice do not edit miR-376 and PRPS1 is increased, suggesting that miRNA editing can alter miRNA target selection *in vivo *[194]. Together, these results indicate that the levels and/or targets of a sub-set of miRNA can be post-transcriptionally regulated by A to I editing.

## Single nucleotide polymorphisms and mutations can alter miRNA processing

Similar to alteration of nucleotides by RNA editing, single nucleotide polymorphisms (SNPs) and point mutations within the pri-miRNA have the potential to alter the secondary structure and prevent processing of the pri-miRNA. An analysis of chronic lymphocytic leukemia pri-miRNA sequences identified mutations in 5 of 42 miRNAs analyzed [36]. While 15% (75 total) of CLL samples exhibited at least one mutation, no mutations were found in 160 normal samples. A germ line cytosine-to-thymine mutation 7 bp 3' to the pre-miR-16-1 region was associated with reduced mature miRNA levels [36]. In addition, a common guanine/cytosine polymorphism within the precursor of miR-146 is associated with increased risk of papillary thyroid cancer [195]. The cytosine allele exhibits reduced expression of mature miR-146 and results in an increase in miR-146 targets compared to individuals with the guanine allele. The SNP is located within the passenger strand of miR-146 and the cytosine allele alters the secondary structure of miR-146 which inhibits Drosha processing [195]. Similarly, a polymorphism within the mature sequence of miR-125a disrupts Drosha processing by introducing a bulge within the hairpin. Processing of miR-125a could be restored by the introduction of a complementary mutation on the passenger strand [196]. Together these studies emphasize the importance of secondary structure in the control of miRNA biogenesis. Despite these examples, polymorphisms which disrupt processing are likely to be rare. Large scale profiling experiments have indicated that most miRNA SNPs occur outside of the pre-miRNA region. Those SNPs which are present within the pre-miRNA are often considered to be neutral because they occur within the loop region or conserve a bulged region [197,198]. This observation may indicate that polymorphisms which alter miRNA processing are detrimental to normal development and have been selected against during evolution.

In addition to altering miRNA biogenesis, SNPs within 3'UTR of target genes may also perturb miRNA function by introducing or disrupting binding sites. Allelic imbalance sequencing of miRNA target genes containing SNPs in the mouse suggests that polymorphic sites may be responsible for the differential regulation of a surprisingly large number of mRNAs (7.5–15%) [199]. Several physiologically relevant examples of altered miRNA targeting due to SNPs have been identified. For example, a SNP within the let-7 binding site of the *KRAS *3'UTR increases the risk of non-small cell lung cancer by reducing let-7-mediated repression of KRAS [200]. Additionally, a SNP within the miR-24 binding site of *dihydrofolate reductase *(DHFR) prevents miR-24 suppression, resulting in DHFR overexpression and resistance to methotrexate based chemotherapy [201]. As the identification of SNPs continues, it will be important to consider the introduction or loss of miRNA targeting sites as potential functional consequences of these small nucleotide changes.

## Regulation of miRNA turnover

While the understanding of mechanisms which control miRNA biogenesis has increased dramatically since the discovery of miRNA, little is known about the mechanisms which control degradation and turnover of miRNAs. In *A. thaliana*, the Small RNA Degrading Nuclease (SDN) proteins degrade single-stranded miRNA, and knockdown of these proteins results in widespread developmental defects [202]. To date, the animal homologues which control miRNA degradation are unidentified. However, *in vivo*, miRNAs may be relatively stable since many miRNAs persist following knockdown of miRNA processing enzymes [203]. These studies also suggest that the degradation of some miRNAs may be differentially regulated, as the degree of reduction following Dicer knockdown varies depending on the miRNA species [203,204]. As in the case of miRNA biogenesis, the degradation of individual miRNA species is likely to be regulated. For example, the stability of mature miR-29b, but not the co-transcribed miR-29a, is modulated in a cell cycle-dependent manner [205]. Extracellular stimuli which modulate the degradation of specific miRNA have also been described. Following hypoxia treatment, mature miR-199a, but not miR-199a* is dramatically reduced in myocytes [206], while in the liver, miR-122 is decreased within one hour of interferon treatment [207]. The identification of factors which regulate both general and specific miRNA turnover represents an important challenge for future studies.

Post-transcriptional modifications of miRNAs may play an additional role in the modulation miRNA turnover. For example, the cytoplasmic poly(A) polymerase GLD-2 catalyzes the addition of a 3'-terminal adenosine following Dicer cleavage and unwinding of miR-122 [208]. Mature miR-122 is reduced in GLD-2 null mice, suggesting that the addition of a 3' adenosine promotes the stability of this miRNA [208]. Large scale sequencing of cloned miRNA often indicates the presence of non-genomically encoded residues on the 3' end of miRNA [163,209]. Furthermore, the addition of either adenosine or uridine has been appreciated to alter the stability of both protein coding and non-coding RNAs, suggesting that this mechanism of miRNA stability regulation may extend beyond miR-122 [210].

## Regulation of miRNA activity

Similarly to the control of total miRNA levels, the activity of miRNA-mediated repression is highly regulated by diverse factors. Following maturation, the mature miRNA is loaded onto Ago proteins and associates with additional proteins to form the RISC complex. While a minimal functional RISC complex can be generated *in vitro *using recombinant Ago2 and small RNA [211]; proteomics approaches have identified dozens of Ago associated proteins, and the differential association of these proteins allows for the modification of RISC activity [212]. For example, members of the TRIM-NHL family of proteins, including TRIM32 in mouse [213], and NHL-2 in *C. elegans *[214], associate with RISC components and enhance the activity of miRNA. Interestingly, sequencing of TRIM32-associated miRNAs indicates that only a subset of miRNAs, including let-7a, interact with TRIM32 (Fig. 3) [213]. In addition to the TRIM-NHL proteins, Importin-8 (Imp8) has also been found to modulate miRNA-medicated silencing through interaction with Agos. Knockdown of Imp8 reduced association of miRNA with Ago2 and abrogated miRNA-mediated repression of endogenous miRNA targets [215]. These findings emphasize that miR-mediated repression could be regulated by alterations in the biogenesis as well as the activity of RISC-associated miRNA. It is also of note that the association of miRNA with specific RISC factors can result in increased translation of target genes [216,217].

The biological function of miRNA can also be altered indirectly by modification of the mRNA target site. For example, mRNA binding factors which interfere with the interaction of miRNA with target sites have been reported. In the liver, cellular stress promotes the association of HuR (ELAV1) with AU-rich elements present in the CAT-1 mRNA [17]. This association antagonizes repression of CAT-1 by miR-122 and allows increased CAT-1 protein levels in response to stress stimuli [17]. miRNA repression of target sites bearing conserved U-rich regions are similarly antagonized by the RNA binding protein Dead end 1 (Dnd1) [218]. These findings suggest that miRNA-mediated repression can be reversible, and the repression of some mRNAs may be context-dependent. Finally, the ability of a miRNA to repress a given mRNA is limited by the presence of miRNA binding sites within the 3'UTR. Alternative usage of cleavage and polyadenylation sites may provide a simple mechanism for the tissue-specific regulation of miRNA-mediated repression. Analysis of alternative 3'UTR isoforms indicates a global reduction of 3'UTR length in proliferating cells [219], while differentiating cells express mRNAs with longer 3'UTRs [220]. As shortened 3'UTRs contain fewer miRNA binding sites, mRNAs in proliferating cells are more resistant to miRNA-dependent repression [219]. It is speculated that the effects of both (i) reduced expression of miRNAs, and (ii) increased expression of mRNAs with shorter 3'UTRs may synergistically contribute to pathogenesis of some human diseases, such as cancer.

## Conclusion

miRNA are generated through the concerted action of multi-subunit complexes which promote the sequential cleavage, export, and loading of miRNA into silencing complexes. An increasing number of reports suggest that each of these steps serves as a potential point of regulation, and therefore provides additional complexity to miRNA-dependent gene regulation. Unlike transcriptional regulation or post-translational regulation of proteins, miRNA is able to modify not only gene expression but also cellular function rapidly in response to environmental cues. In particular, regulation of miRNA biogenesis may serve as the first line of response following stimulation to promote the modulation of gene expression programs. Many of the mechanisms of miRNA regulation are limited to a particular set of miRNA which could allow the co-regulation miRNA in response to cellular stimuli. As a single miRNA modulates the expression of many targets simultaneously, the co-regulation of several miRNAs could have a dramatic impact on gene expression and cellular physiology. In the future it will be important to determine the mechanisms of miRNA co-regulation, as well as the influence of such co-regulated miRNAs on cellular processes.

As the altered expression of miRNAs has been implicated in various human diseases, small molecules that modulate specific miRNA expression or activity are attractive candidates for the therapeutic intervention of certain diseases. For example, a small molecule screen for enhanced silencing by miRNAs yielded a quinalone called Enoxacin which increases the affinity of TRBP for RNA and promotes RISC loading [221]. While Enoxacin may increase the activity of miRNA in non-specific manner, a more detailed understanding of mechanisms of miRNA regulation may allow for the identification of small molecules which specifically target a set of miRNAs. As more mechanisms of microRNA regulation are uncovered, an important challenge will be the elucidation of how multiple mechanisms of miRNA regulation cooperate to orchestrate the fine tuning of miRNA expression and function.

## Competing interests

The authors declare that they have no competing interests.

## Authors' contributions

BD and AH wrote the manuscript. All authors read and approved the final manuscript.
